# C8-ceramide modulates microglia BDNF expression to alleviate postoperative cognition dysfunction via PKCδ/NF-κB signaling pathway

**DOI:** 10.1007/s00221-024-06847-2

**Published:** 2024-05-15

**Authors:** Guangqian Li, Yuhao Wang, Lei Qian, Danni Li, Yuchen Yao, Jian Pan, Dan Fan

**Affiliations:** 1 Department of Anesthesiology, Sichuan Provincial People’s Hospital, University of Electronic Science and Technology of China, #32 West Second Section, First-Ring Road, Chengdu, 610072 People’s Republic of China; 2 State Key Laboratory of Oral Diseases and National Center for Stomatology and National Clinical Research Center for Oral Diseases and Department of Oral and Maxillofacial Surgery, West China Hospital of Stomatology, Sichuan University, Chengdu, 610041 Sichuan China; 3 Frontier Innovation Center for Dental Medicine Plus, West China Hospital of Stomatology, Sichuan University, Chengdu, 610041 Sichuan China

**Keywords:** C8-ceramide, POCD, Microglia, BDNF, PKCδ/NF-κB signaling pathway

## Abstract

Postoperative cognitive dysfunction (POCD) is a kind of serious postoperative complication in surgery with general anesthesia and it may affect patients’ normal lives. Activated microglia are thought to be one of the key factors in the regulation of POCD process. Once activated, resident microglia change their phenotype and secrete kinds of cytokines to regulate inflammatory response in tissues. Among these secretory factors, brain-derived neurotrophic factor (BDNF) is considered to be able to inhibit inflammation response and protect nervous system. Therefore, the enhancement of BDNF expression derived from resident microglia is suggested to be potential treatment for POCD. In our study, we focused on the role of C8-ceramide (a kind of interventional drug) and assessed its regulatory effect on improving the expression of BDNF secreted from microglia to treat POCD. According to the results of our study, we observed that C8-ceramide stimulated primary microglia to up-regulate the expression of BDNF mRNA after being treated with lipopolysaccharide (LPS) in vitro. We proved that C8-ceramide had ability to effectively improve POCD of mice after being accepted carotid artery exposure and their abnormal behavior recovered better than that of mice from the surgery group. Furthermore, we also demonstrated that C8-ceramide enhanced the cognitive function of mice via the PKCδ/NF-κB signaling pathway. In general, our study has confirmed a potential molecular mechanism that led to the occurrence of POCD caused by surgery and provided a new clinical strategy to treat POCD.

## Introduction

POCD is a kind of serious complication after surgery with general anesthesia. People who suffer from POCD always have persistent disturbances in memory, delirium, poor comprehension and inattention in social activities (Krenk et al. 2010; Monk and Price 2011; Yang et al. 2022). Millions of patients worldwide accept surgery with general anesthesia every year, so the potential population with surgery-induced POCD cannot be ignored. The previous studies have showed that the incidence of POCD was as high as 25.8% on the 1st week after surgery and it was still 10% on the 3rd month after surgery (Moller et al. 1998). Severe POCD may prolong patients’ hospital stay, seriously affect their life quality, and also lead to high mortality rate (Steinmetz et al. 2009). POCD will cause new pain to patients after surgery and bring a heavy burden to society and medical insurance (Fondeur et al. 2022). How to prevent and treat POCD has gradually become important and necessary to academic circles in recent years.

In previous studies, scholars have thought that the occurrence of POCD was induced by an extremely complex molecular network. Among them, inflammatory response, oxidative stress and neurotrophic factor deprivation are important to the process of POCD (Golia et al. 2019; Saxena et al. 2019). It should be pointed out that the neuroinflammation caused by surgery has been widely accepted to be able to induce POCD (Alam et al. 2018; Kawano et al. 2018). Many previous studies have showed that the occurrence of POCD was often accompanied by a significant increase in the expression of inflammatory factors in the nervous system, which has suggested that neuro-inflammation regulatory should receive broader attention in the treatment of POCD (Lin et al. 2020; Su et al. 2020). The activation of the peripheral immune system after inhalation general anesthesia can enhance the expression of inflammatory factors and the accumulation of reactive oxygen species in the central nervous system (CNS), which will further lead to the activation of microglia (Saxena et al. 2019; Hovens et al. 2015; Goehler et al. 2000).

Microglia are the resident immune cells originating in the yolk sac in the CNS and they have ability to migrate into the brain at the early stage of life (Degos et al. 2013). Microglia are widely distributed in the CNS and they regulate the stress response in certain circumstances (Askew et al. 2017). In the previous studies, activated microglia had ability to release pro-inflammatory factors and reactive oxygen compounds, which led to the occurrence of POCD (Saxena et al. 2019). During the inflammatory process, inflammatory related signaling pathways are activated, which improves the secretion of pro-inflammatory factors derived from immune cells and induces the process of tissue inflammatory. The PKCδ/NF-κB signaling pathway has been considered as a classical pro-inflammatory signaling pathway. The activation of NF-κB up-regulate the expression of tumor necrosis factor ɑ (TNFɑ), interleukin-1 (IL-1), IL-6, IL-8 and adhesion molecules in the tissues (Hoesel and Schmid 2013). The pro-inflammatory factors are able to accelerate the aggregation of leukocytes and further enhance tissue inflammation. The activation of NF-κB is a phosphorylation process under the regulation of kinds of kinases, such as mitogen-activated protein kinase family (MAPK), protein kinase C (PKC) and Akt (Chen et al. 2023; Dan et al. 2008; Moscat et al. 2003). Among them, PKCδ in the PKC family is important to effectively phosphorylate NF-κB (the signal to activate the PKCδ/NF-κB signaling pathway) and stimulate immune cascade reactions.

The role of microglia in the stress response depends on the fact that they not only secrete inflammatory factors to damage tissues, but also secrete neurotrophic factors (such as BDNF) to repair injured tissues (Parkhurst et al. 2013). BDNF belongs to neurotrophin family, which is able to induce the neural differentiation of stem cells and has anti-inflammatory effects to improve the microenvironment in the CNS (Ge et al. 2019; Wang et al. 2019). BDNF determines the neuron-glia communication and maintain the homeostasis in the nervous system (Fields and Burnstock 2006; Takasaki et al. 2008). In the inflammatory tissues, pro-inflammatory factors directly damage neurons’ function and synaptic plasticity, and then microglia receive the signals from injured neurons to up-regulate the expression of BDNF (Gómez-Casati et al. 2010). In this process, the role of the BDNF/TrkB signaling pathway is thought to be important in the secretion of BDNF, and several previous studies have found the activation of the BNDF/TrkB signaling pathway was able to enhance the expression level of BDNF derived from the microglia (Lee et al. 2010). Some other studies have also showed that the improving expression of BDNF had positive impact on patients’ cognitive function in a certain degree after surgery (Fan et al. 2016; Calabrese et al. 2014). In general, how to enhance the concentration of BDNF in the CNS may become a potential therapeutic strategy to POCD.

Ceramide family contains kinds of drugs acting as lipid second messengers and they are involved in various cellular activities, including cell growth, differentiation and inflammation (Singh et al. 2012). C8-ceramide is a synthetic cell-permeable short chain ceramide analog and it is used in studies to explore the pharmacological effect of nature ceramide (Hamanaka et al. 2002). A previous study has demonstrated that ceramide could decrease the concentration of NO and PGE_2_ induced by LPS via the inhibition of NF-κB, AP-1and PKC (Hsu et al. 2001). C8-ceramide has been proved to have ability to reduce the expression of IL-6 and TNF-α derived from activated macrophages (Chiba et al. 2007). Some scholars pointed out in their studies that the anti-inflammatory effect of exogenous ceramide on microglia may be the result of their inhibitory regulation on the MAPKs, PI3K/Akt and Jak/STAT signaling pathways (Jung et al. 2013). Furthermore, Nakajima et al. have demonstrated that C8-ceramide could enhance the expression of BDNF derived from microglia (Nakajima et al. 2002).

Therefore, we hypothesized that C8-ceramide had ability to play protective effect on cognitive function via promoting the expression of BDNF and suppressing neuro-inflammation. In this study, primary microglia were stimulated by LPS and C57BL/6J mice were subjected to right carotid artery exposure. The expression of BDNF and pro-inflammatory cytokines (IL-6, TNF-α) was assessed at different time after surgery. The learning and memory of mice in all groups were evaluated by the Morris Water Maze (MWM) test and fear conditioning test. The expression level of related signaling pathway proteins in hippocampus of mice were tested by western blot and the polarization of microglia was detected by immunofluorescence staining.

## Methods

### Cell culture

Microglia were harvested from C57BL/6J mice as previously described (Chhor et al. 2013). In brief, C57BL/6J mice (within one day after birth) were soaked into 75% ethanol approximately 10 s and then dissected their cortices in D-Hank’s balanced salt solution with 2% penicillin–streptomycin (PS, Gibco, USA). The cortices were quickly moved into centrifuge tube containing pre-cooled high glucose Dulbecco’s modified Eagle’s medium (DMEM, Gibco, USA) supplemented with 10% fetal bovine serum (FBS, Gibco, USA) and 1% PS, and then dissociated them into small pieces for later using. The cell suspension was keep in culture incubator (37 ℃, 5% CO_2_). Microglia were isolated from mixed glial cells on the 12th to 14th day. Microglia were collected after centrifugation (1000 rpm × 15 min) and replated in culture dishes containing DEME with 10% FBS at a density of 7.5 × 10^5^ cells/ml. The purity of microglia was revealed by immunofluorescence staining by testing the expression of Iba-1 (the specific marker to microglia) (n = 3). The harvested microglia were be divided into the following four groups: the control group, the LPS treated group, the LPS + C8-ceramide treated group and the C8-ceramide treated group. The mice would accept C8-ceramide (25 μM) treatment 30 min before LPS intervention (1 μg/ml). The total RNA was collected at the 24th hour after treatment to test the expression of BDNF mRNA in all four groups.

### Animals and surgery

The eight-weeks-male C57BL/6J mice (24–28 g) were purchased from Chengdu Dossy experimental animal company, LTD. Before the surgery, all mice were given sufficient food and water in the comfortable and stable environment (12 h light/dark cycle; 24 ± 1 ℃ room temperature; 55 ± 10% relative humidity) to adapt to the new environment. All mice were randomly divided into four groups: the control group, the surgery group, the surgery + C8-ceramide group and the C8-ceramide group. There were 43 mice in each group. The mice in the surgery + C8-ceramide group and the C8-ceramide group were treated with C8-ceramide at the dosage of 10 mg/kg (0.1 ml per mouse) through intraperitoneal injection for 4 consecutive days before being subjected to surgery (Ponnapakkam et al. 2018; Balakrishnan et al. 2016).

In our previous study, the right carotid artery exposure surgery was used to construct animal POCD disease model (Fan et al. 2016). In the operation, the mice were monitored and stayed on a heating blanket at a stable temperature of 37 ℃. A 1.5 cm-length incision was made in the central of neck after 30-min sevoflurane inhalation. The soft tissue covering the trachea was gently removed to expose the common carotid artery. The common carotid artery was completely separated and explored without injuring the vagus nerve. Then, the wound was disinfected and sutured carefully. The operation was performed in a sterile environment and lasted approximately 10 min. The mice were finally anesthetized by 3% sevoflurane inhalation for 2 h. All animals were put back into their own cages after awakening.

### Behavioral tests

#### MWM test

The mice in all groups were tested by the MWM test to assess their spatial memory ability as previously described (Vorhees and Williams 2006). The MWM test chamber was a circular tank with a 122 cm diameter and a 50 cm height, and there was a submerged platform placed 1 cm below the water surface. The temperature and humidity in the MWM test room were constant and the water temperature was keep in 24 ± 1℃. It must be pointed out that all things in the test room were stayed in the same place without any movement throughout the test period.

The MWM test chamber was equally divided into four quadrants and the submerged platform quadrant located in one of quadrants, which was called the target quadrant. The swimming path of mice was recorded by the WMT-100s water maze video analysis system. In the training period, mice in all groups were given a five-day training, which contained 4 trials per day and 60 s per trial (the time interval between two trials was 30 min). Once the mice stayed on the platform for at least 15 s, the system would stop timing and it was defined as the escape latency period. In the testing period, the mice were placed at the opposite side of the target quadrant and the hidden platform was removed. The results were shown as how many times mice passed through the platform and how long they stayed in the target quadrant within 60 s.

#### Fear conditioning test

The fear conditioning test included the context-related fear test and tone-related fear test, and these two parts were able to test hippocampal-dependent and non-hippocampal-dependent cognitive function respectively. The previous studies about the fear conditioning test were referred in our experiment design (Zhang et al. 2014). In our study, the training began on the 13th day after surgery and test at 24 h after the training (Wang et al. 2023.) The mice in all groups were placed in a dark test chamber which was wiped with 70% alcohol in advance, and then they were stimulated by three tone-foot shock pairings (tone 2000 Hz, 85 db, 30 s; foot shock 0.7 mA, 2 s) with 1-min time interval. The context-related test started at the 24th hour after training. Then, mice were put back to the same test chamber for 8 min without any shock and recorded their freezing behavior. Mice were placed in a relatively light chamber, where was wiped with H_2_O_2_ in advance and the tone stimulation (2000 Hz, 85 db, 30 s) was turned on for three cycles with 1-min time interval. All mouse freezing behavior was recorded by a camera and their freezing time was used to evaluate their memory function.

### Blood and brain tissue harvesting

Mice in all groups were anesthetized with sevoflurane and their thoracic cavity was opened to expose the heart. The blood samples of mice in each group were collected directly from their hearts. All mice were perfused with ice-cold normal saline. The hippocampus of mice in all groups were isolated immediately and stored in liquid nitrogen. The brain tissues were fixed in 4% paraformaldehyde and coronary artery was take out for immunofluorescence staining. The blood samples were centrifuged for 15 min at the speed of 1500 rpm and the plasma was collected. The hippocampus of mice were homogenized in cold RIPA buffer containing enzyme inhibitor and then the solution was centrifuged at the speed of 12,000 rpm for 15 min. Finally, the supernates were collected and stored at  − 80 ℃ for future use.

### Enzyme-linked immunosorbent assay (ELISA) analysis

The concentration of TNF-α and IL-6 were tested at the 6th and 24th hours after surgery. After being homogenized and centrifuged, the concentration of protein collected from mouse hippocampus in each group was calculated by bicinchoninic acid (BCA) assay. The expression level of TNF-α and IL-6 in plasma and hippocampus were measured by TNF-α ELISA kits (R&D systems, USA) and IL-6 ELISA kits (R&D systems, USA) according to the operation instruction.

### Western blot

The hippocampus of mice in each group was collected on the 3rd and 7th days after surgery for western blot. The protein samples were boiled in 5* loading buffer and separated by sodium dodecyl sulfate–polyacrylamide gel electrophoresis (SDS-PAGE). Then the protein on SDS-PAGE was transferred to poly-(vinylidene fluoride) (PVDF) membranes. PVDF membranes were blocked with 5% milk for 90 min at room temperature and incubated with primary antibodies at 4 ℃ overnight. PVDF membranes were washed with TBST buffer for three times and then incubated with secondary antibodies for 2 h at room temperature on the next day. The protein bands on PVDF membranes were visualized in chemiluminescence machine (Bio-Rad, USA) with using enhanced chemiluminescence kit (Solarbio, China).

The primary antibodies used in this study were showed as follows: anti-BDNF antibody (1:1000 dilution, Abacm, UK); anti-phospho-TrkB (Y705) antibody (1:1000 dilution, Abacm, UK); anti-TrkB antibody (1:1000 dilution, Abacm, UK); anti-PKCδ antibody (1:1000 dilution, Cell Signaling Technology, USA); anti-phospho-PKCδ antibody (1:1000 dilution, Cell Signaling Technology, USA); anti-NF-κB p65 antibody (1:1000 dilution, Cell Signaling Technology, USA); anti-iNOS antibody (1:1000 dilution, Cell Signaling Technology, USA); anti-Arg-1 antibody (1:1000 dilution, Cell Signaling Technology, USA); anti-Tubulin antibody (1:2000 dilution, Cell Signaling Technology, USA); anti-β-actin antibody (1:2000 dilution, Cell Signaling Technology, USA). The β-actin and tubulin were used as the reference proteins.

### Immunofluorescent staining

To observe the activation of microglia, mice were sacrificed to harvest brain tissues at the 24th hour after surgery. Brain tissues were fixed in 4% paraformaldehyde at room temperature for three days and embedded in paraffin. After being repaired by EDTA, the sections were washed with PBS buffer and blocked with 5% bovine serum albumin at room temperature for 1 h. The sections were incubated with primary antibody overnight at 4 ℃ and then washed with PBS buffer for three times. The secondary antibodies were added to cover the sections completely at room temperature for 1.5 h in the dark. After being washed with PBS buffer, sections were stained with 4′,6-diamidino-2-phenylindole (DAPI) to locate nucleus for 5 min and immediately sealed with anti-fluorescence quenching agent. Stained images were obtained through the LSM700 confocal microscopy system (ZEISS, Germany).

The primary antibodies used in this study are shown as follows: anti-Iba-1 antibody (1:200 dilution, Abacm, UK); anti-iNOS antibody (1:200 dilution, Cell Signaling Technology, USA); anti-Arg-1 antibody (1:200 dilution, Cell Signaling Technology, USA).

### Reverse transcription polymerase chain reaction (RT-PCR)

The total RNA of microglia with different intervention was collected with Trizol reagent (Sigma-Aldrich, USA) according to the operation instruction. The concentration and purity of total RNA were measured by using Microvolume UV–Vis Spectrophotometer (Thermo, Germany). Then, the reverse transcription of the total RNA was completed by using the HiScript II Q RT SuperMix for qPCR kit (Vazyme Biotech, China). The synthesized cDNA templates were used to do quantitative real-time PCR by using SYBR Green PCR reagents (Bio-Rad, USA). The ΔΔCt (the threshold cycle) values were calculated and the results were expressed as the ratio of the mRNA copies of BDNF gene to that of 18s RNA gene (reference gene). All data was presented in the fold change compared to the control group.

The primers involved in our study were showed as follows: 5′-TTACCTGGATGCCGCAAACAT-3′ (forward) and 5′-TGACCCACTCGCTAATACTGTC-3′ (reverse) for BDNF; 5′-CGGACAGGATTGACAGATT-3′ (forward) and 5′-CAAATCGCTCCAACCAACTAA-3′ (reverse) for 18s RNA.

### Statistic analysis

Data was showed as mean ± standard deviation (n ≥ 3). The homogeneity variance was tested and all data was analyzed by one-way analysis of variance (ANOVA using the SPSS 22.0 software). Fisher’s Least Significant Difference (LSD) test was used for post-hoc analysis. All statistical graphs in this paper were performed by Graphpad prism 6.0 system. The *p*-value less than 0.05 was considered to be significant difference.

## Results

### C8-ceramide promoted BDNF expression in microglia and attenuated learning and memory impairment after surgical trauma

**Fig. 1:**
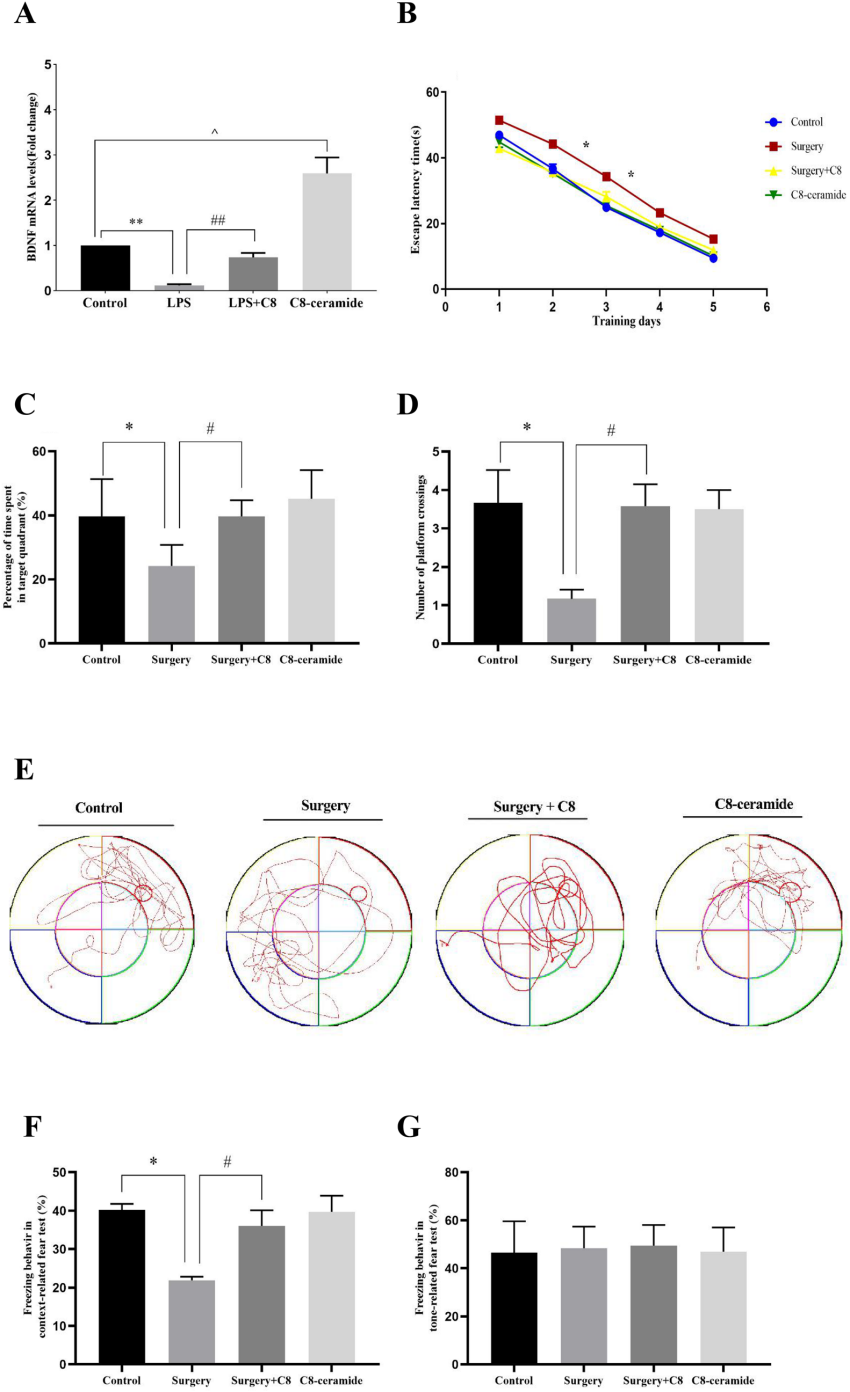
The results of the RT-PCR and behavioral tests. (**A)** The expression of BDNF mRNA of primary microglia in each group. (**B**) The escape latency time of mice during the 5-days training. (**C**) The percentage of time spent in target quadrant of mice in each group. (**D**) The number of platform crossings of mice in each group. (**E**) The swimming path of mice recorded by a video camera. (**F**) The freezing time in contextual test. (**G**) The freezing time in tone test. (* *p* < 0.05, compared with the control group; # *p* < 0.05, compared with the surgery + C8-ceramide group; ^ *p* < 0.05, the C8 group compared with the control group, n = 43)

To investigate the effect of C8-ceramide on the BDNF expression derived from microglia in inflammatory tissues, primary microglia were treated with C8-ceramide before LPS exposure and then assessed the expression of BDNF mRNA by RT-PCR. As the results showed, the expression of BDNF mRNA in the LPS-treated group were significantly down-regulated than that in the control group (Fig. 1A). However, when primary microglia were pre-treated with C8-ceramide, the reduction of BDNF mRNA expression mRNA induced by LPS would be reversed and return to the same expression level with the control group. Furthermore, C8-ceramide used alone was able to significantly increased the expression of BDNF mRNA compared to those of mice from other groups. The above data strongly indicated that C8-ceramide could stimulate primary microglia to secrete BDNF and avoid damage to the nervous system caused by inflammation response.

In order to explore whether C8-ceramide had effect on surgery-induced cognitive dysfunction, the MWM test and the Fear conditioning test were used to evaluated mice’ learning and memory ability. At first, all mice were subjected to train for five days on the 7th day after surgery. Compared to the mice in the control group, mice accepting surgery were significantly increased their latency to find the hidden platform on the 2nd and 3rd day in the training process (Fig. 1B). However, mice pre-treated with C8-ceramide have effectively restored the latency to find hidden platform and no significant differences could be found when compared to that in the control group. As the training time increasing, the escape latency period of the mice in each group gradually shortened, which indicated that the mice in all groups had ability to find the hidden platform and could further conduct the MWM test (Fig. 1B). On the 6th day in the spatial probe test, the swimming path recorded by computer showed that mice in the surgery group spent the least time in the target quadrant and cross the original hidden platform infrequently when compared to the mice in the other groups (Fig. 1C–E). Furthermore, when mice were pre-treated with C8-ceramide before the surgery, their learning and memory ability was same with the mice in the control group, which demonstrated that C8-ceramide had ability to protect the hippocampus of mice and prevent the process of POCD induced by surgery.

Similar to the MWM test, the context-related freezing time of mice in the surgery group was significantly decreased than those of other groups, and their cognitive impairment could be attenuated by the pre-treatment of C8-ceramide (Fig. 1F). It must be pointed out that there had no significant difference among all four groups in the tone-related fear test (Fig. 1G). It indicated that the surgery-induced impairment of hippocampus-dependent cognitive function could be restored by using C8-ceramide and surgery could not cause damage to the non-hippocampus-dependent cognitive function.

### C8-ceramide reversed the down-regulation of BDNF expression induced by surgery

**Fig. 2:**
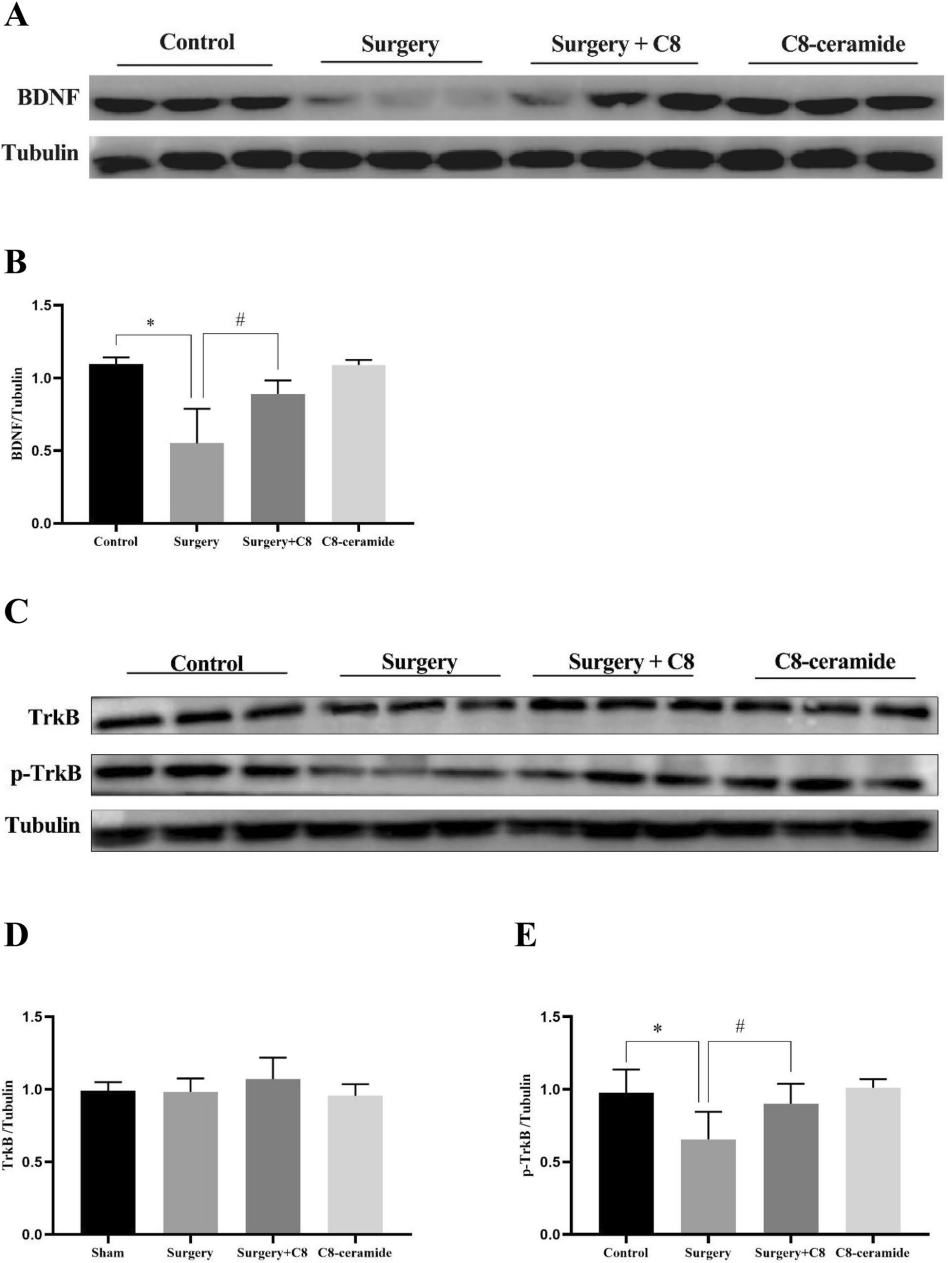
The expression of the BNDF/TrkB signaling pathway in the hippocampus of mice in all groups at the 3rd day after surgery. (**A**, **B**) The expression of BDNF in the hippocampus of mice in all groups at the 3rd day after surgery. (**C**–**E**) The expression of total TrkB and p-TrkB in the hippocampus of mice in all groups at the 3rd day after surgery. (* p < 0.05, compared with the control group; # p < 0.05, compared with the surgery + C8-ceramide group, n = 3)

In the previous study, we verified that C8-ceramide enhanced the secretion of BDNF derived from primary microglia, which could reverse the effect of LPS in vitro. To explore whether C8-ceramide have the same effect on microglia in vivo and the potential molecular mechanism in these process, the hippocampus in mice from all groups was collected on the 3rd and 7th day after surgery. The results of western blot showed that surgical procedures had negative effect on the expression of BDNF in the hippocampus of mice on the 3rd day, but this inhibition effect would be reversed when C8-ceramide was used in advance (Fig. 2A, B). However, using C8-ceramide alone did not improve the expression of BDNF in the hippocampus of mice when compared to the control group.

Due to the fact that the BNDF/TrkB signaling pathway has been considered as the classical signaling pathway to regulate the secretion of BDNF, we assumed that C8-ceramide could up-regulate the expression of BDNF via activating the BNDF/TrkB signaling pathway. In our study, the total expression of TrkB did not have any significant difference in all four groups, but the p-TrkB expression level in the surgery group was the lowest compared to those of other groups (Fig. 2C–E). Consistent with the trend of BDNF expression, using C8-ceramide alone did not significantly increase the expression of p-TrkB, which indicated that C8-ceramide, as a neurotrophic substance, may only promote the secretion of BDNF under stress condition to protect the nervous system. The above data has demonstrated that C8-ceramide promoted the secretion of BDNF via up-regulating the BNDF/TrkB signaling pathway in surgery-induced cognitive dysfunction.

**Fig. 3:**
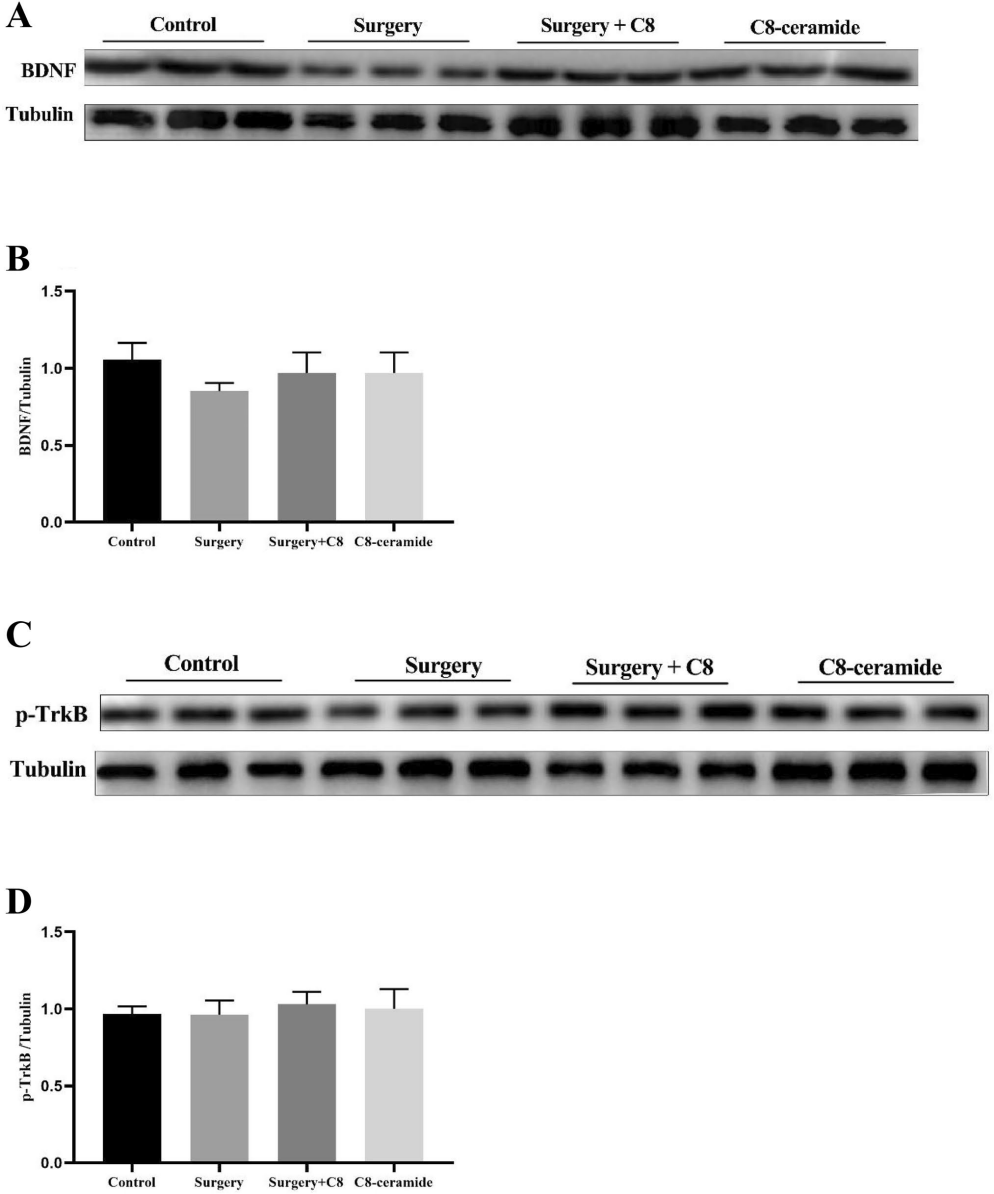
The expression of the BNDF/TrkB signaling pathway in the hippocampus of mice in all groups at the 7th day after surgery. (**A**, **B**) The expression of BDNF in the hippocampus of mice in all groups at the 7th day after surgery. (**C**, **D**) The expression of p-TrkB in the hippocampus of mice in all groups at the 3rd day after surgery (n = 3)

However, we also found that there had no significant difference in both the expression of BDNF and p-TrkB in the hippocampus of mice from all the groups on the 7th day after surgery (Fig. 3A–D). It suggested that C8-ceramide could treat surgery-induced cognitive impairment at the early stage after surgery and shorten the duration of POCD to enhance the life quality of patients.

### C8-ceramide down-regulated the expression of surgery-induced pro-inflammatory factors in the hippocampus of mice

**Fig. 4:**
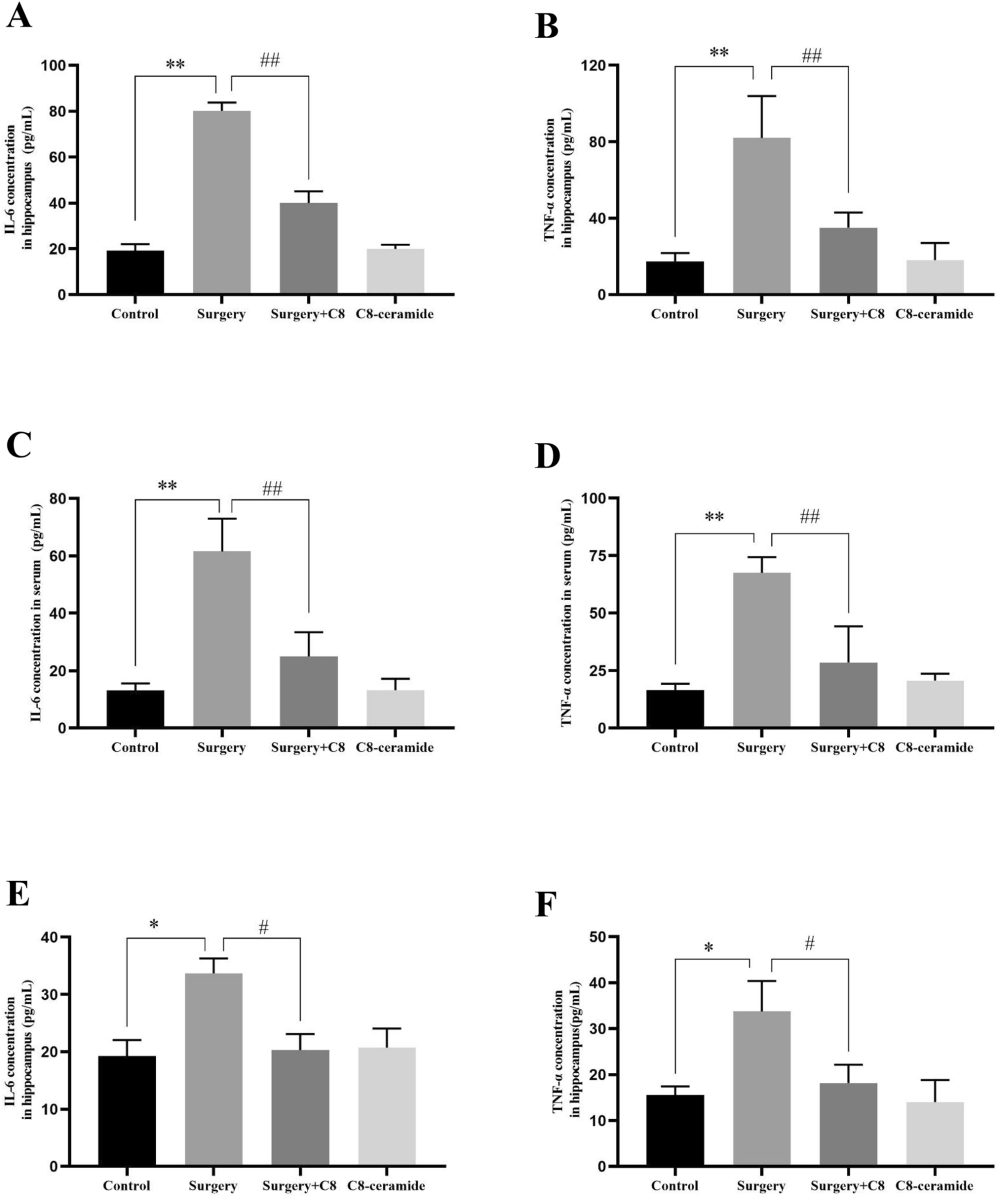
The inflammation level in the hippocampus and serum of mice in all groups at the 6th and 24th hour after surgery. (**A**, **B**) The expression of inflammatory factors in the hippocampus of mice in all groups at the 6th hour after surgery. (**C**, **D**) The expression of inflammatory factors in the serum of mice in all groups at the 6th hour after surgery. (**E**, **F**) The expression of inflammatory factors in the hippocampus of mice in all groups at the 24th hour after surgery. (* p < 0.05, compared with the control group; # p < 0.05, compared with the surgery + C8-ceramide group; ** p < 0.01, compared with the control group; ## p < 0.01, compared the surgery + C8-ceramide group, n = 3)

Although the entire surgical process in our study was in a sterile environment, other predispositions could cause tissue inflammation, including surgical trauma. To evaluate the relationship between POCD and tissue inflammation, as well as the role of C8-ceramide on the inhibition of tissue inflammation, the blood serum and hippocampus of mice in all groups were harvested to test the concentration of TNF-α and IL-6. Compared with the control group, the expression of IL-6 and TNF-α were significantly increased in the hippocampus of mice on the 6th hour after surgery (Fig. 4A, B). Meanwhile, the similar expression change of these two pro-inflammatory factors was also found in the collected serum in the surgery group (Fig. 4C, D). However, when mice were pre-treated with C8-ceramide, the concentration of TNF-α and IL-6 in the hippocampus and serum of mice was close to that in the control group, which suggested that the increasing expression of BDNF induced by C8-ceramide inhibited the surgery-induced inflammatory response in the hippocampus of mice. Furthermore, it must be pointed out that the expression of IL-6 and TNF-α in the hippocampus of mice from the surgery group was still the highest in all four groups at the 24th hour after surgery and it meant that even for a long period of time after surgery, the hippocampus of mice in the surgery group was still in the inflammatory state and it was clearly detrimental to the development of POCD (Fig. 4E, F).

### C8-ceramide inhibited the PKCδ/NF-κB signaling pathway

NF-κB is an important transcription factor to regulate the expression of various inflammatory cytokines. The activation of NF-κB requires the phosphorylation process and it can be stimulated by kinds of proteins and transcription factors, including PKCδ. As the previous studies showed, the PKCδ/NF-κB signaling pathway was important in inflammatory response. Therefore, to test whether the PKCδ/NF-κB signaling pathway was activated in the hippocampus of mice to induce the inflammatory response, we detected the expression of key proteins in this signaling pathway.

**Fig. 5:**
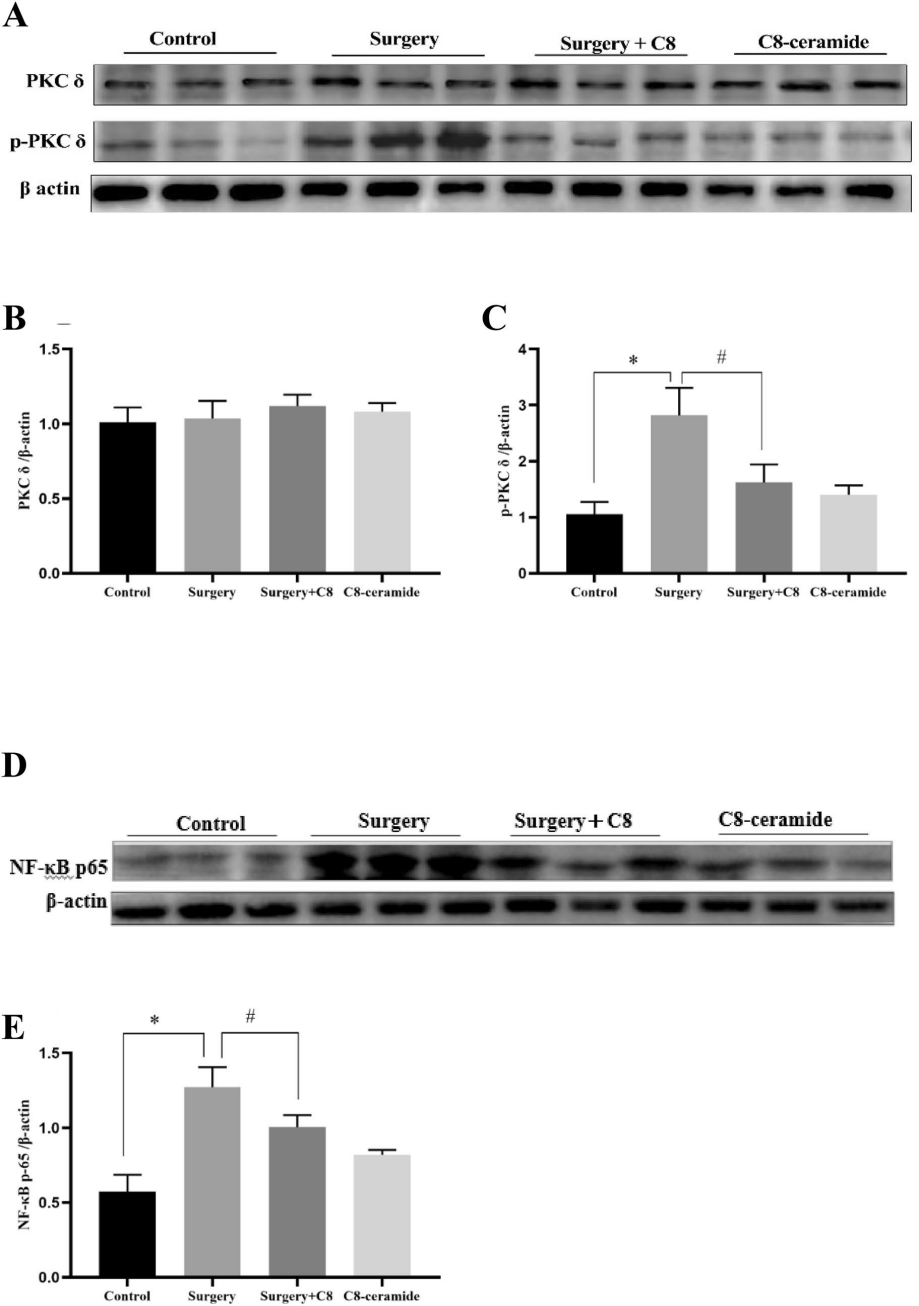
C8-ceramide inhibited the the activation of the PKCδ/NF-κB pathway in the hippocampus of mice. (**A**–**C**) The expression of total PKCδ and p-PKCδ in the hippocampus of mice in all groups. (**D**, **E**) The expression of p-p65 in the hippocampus of mice in all groups. (* p < 0.05, compared with the control group; # p < 0.05, compared with the surgery + C8-ceramide group, n = 3)

In our study, we found that the total expression of PKCδ in the hippocampus of mice from all four groups was close and there were no significant difference among them, but the phosphorylation of PKCδ was significantly enhanced in the hippocampus of mice from the surgery group (Fig. 5A–C). As the phosphorylation level of PKCδ increased, we also found that the phosphorylation of NF-κB (p65) also enhanced in the hippocampus of mice from the surgery group, which indicated that the PKCδ/NF-κB signaling pathway was activated in the hippocampus (Fig. 5D, E). Interestingly, when mice were pre-treated with C8-ceramide before surgery, the phosphorylation process of PKCδ and NF-κB in their hippocampus was inhibited. The above data and the previous Elisa tests on TNF-α and IL-6 confirmed with each other, which indicated that the cognitive dysfunction caused by surgery was related to the activation of the PKCδ/NF-κB signaling pathway and the enhancement of BDNF expression derived from microglia had ability to suppress the surgery-induced inflammation in the hippocampus of mice.

### C8-ceramide had ability to promote the activation of microglia to M2 phenotype

**Fig. 6:**
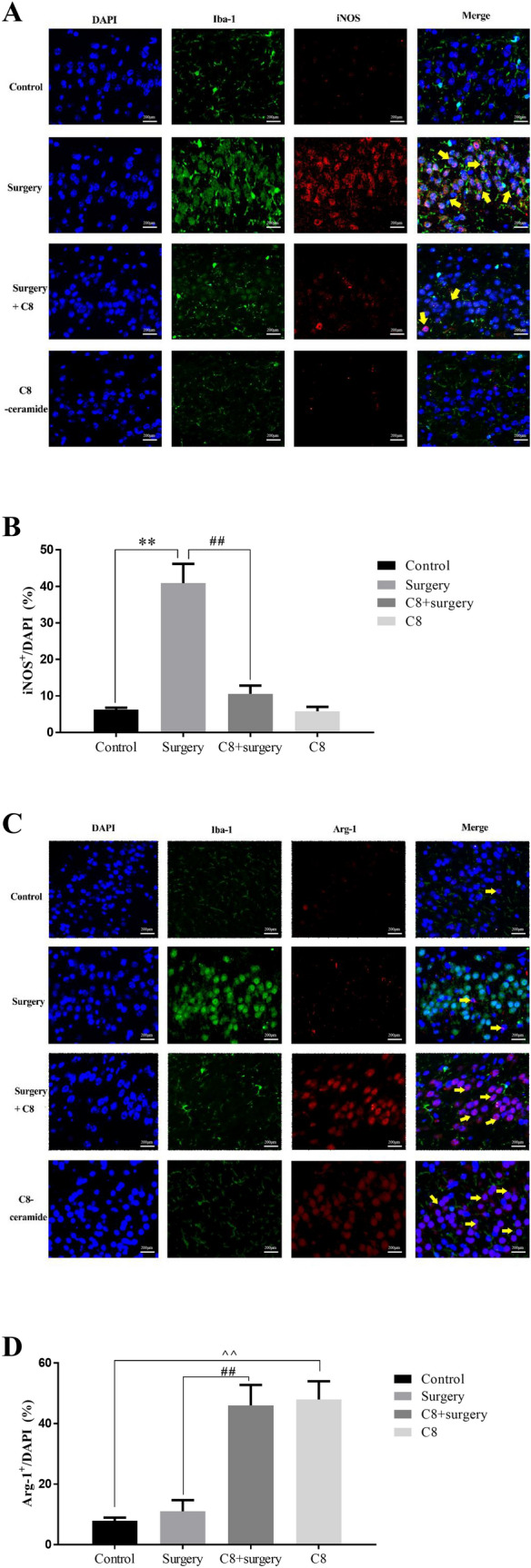
The immunofluorescence of the polarization of microglia in the hippocampus of mice in all groups. (**A**, **B**) The density of M1 microglia in the hippocampus of mice in all groups. (**C**, **D**) The density of M2 microglia in the hippocampus of mice in all groups. Scale bars were 200 μm. (**/^^ p < 0.01, compared with the control group; ## p < 0.01, compared with the surgery + C8-ceramide group, n = 3)

The process of tissue inflammation is determined by the polarization of macrophages. As macrophages in the CNS, the polarization of microglia in the hippocampus plays a decisive role on the process of POCD. To test the polarization of microglia in the hippocampus of mice, we used immunofluorescence staining to detect the expression of specific antigen of microglia. iNOS and Arg-1 were recognized as the specific markers to M1/M2 microglia respectively. The results showed that there were more M1 microglia (iNOS positive cells) in the hippocampus of mice in the surgery group than those of other groups (Fig. 6A, B). On the contrast, M2 microglia (Arg-1 positive cells) mainly existed in the hippocampus of mice from the C8-ceramide group (Fig. 6C, D). The density of M1 and M2 phenotype of microglia regulate the process of inflammatory response in the hippocampus of mice and it was also consistent with the previous study findings.

**Fig. 7:**
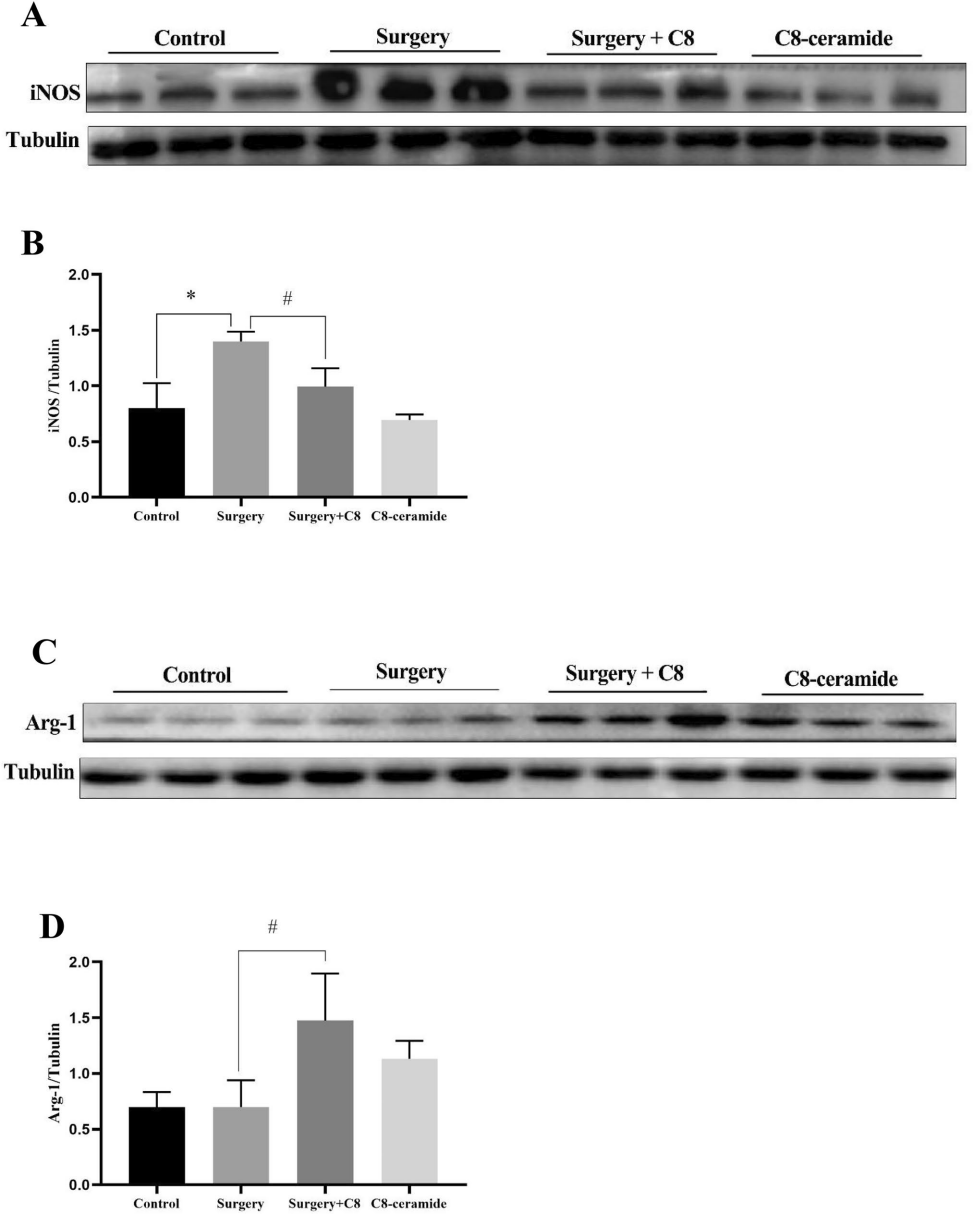
The expression of specific protein to identify the polarization of microglia. (**A**, **B**) The expression of iNOS in the microglia from the hippocampus. (**C**, **D**) The expression of Arg-1 in the microglia from the hippocampus. (* p < 0.05, compared with the control group; # p < 0.05, compared with the surgery + C8-ceramide group; ^ p < 0.05, the C8 group compared with the control group, n = 3)

Furthermore, we found that the expression of iNOS and Arg-1 in the hippocampus of mice were different in all four groups tested by western blot. In the surgery group, the expression of iNOS was significantly higher than that of other groups (Fig. 7A, B). Once mice were pre-treated with C8-ceramide, the expression of Arg-1 would be enhanced and showed that the increasing number of M2 microglia (Fig. 7C, D). The above data suggested that C8-ceramide had ability to regulate the polarization of microglia to surppress the inflammatory response.

## Discussion

In our study, surgery process could cause the cognitive dysfunction and down-regulate the expression of BDNF in the hippocampus of mice. Meanwhile, we also found that the hippocampus of mice in the surgery group was in a high inflammatory state, which was supported by the enhancement concentration of pro-inflammatory cytokines and the density of M1 microglia in the mice’ hippocampus. The application of C8-ceramide reversed the inflammatory response in the hippocampus of mice and stimulated the polarization of microglia towards M2 phenotype in our study. LPS is a kind of gram-negative cell wall component to induce the strong inflammatory response through TLR4 signaling pathway and significantly decrease the expression of BDNF mRNA in primary microglia in vitro (Rahimifard et al. 2017). LPS is often used in vitro to simulate the inflammatory response in vivo. Our research findings have provided strong evidences to show that surgery-induced cognitive dysfunction was the outcome of uncontrolled inflammation response and the decreasing expression of BDNF in the hippocampus of mice.

Many previous studies have demonstrated that surgery-induced neuro-inflammation was an independent risk factor in the development of POCD (Su et al. 2020; Huang et al. 2018). In our study, TNF-α and IL-6 were used as the representative cytokines to assess the inflammatory response. The concentration of TNF-α and IL-6 were significantly increased in the serum and hippocampus of mice in the surgery group at the 6th and 24th hours after surgery. We measured the concentration of pro-inflammatory factors in serum and hippocampus at the 6th hours post surgery, while only pro-inflammatory factor concentration in hippocampus was measured at the 24th hours post surgery. The reason was that we thought that surgical procedures tor mice were limited to brain tissues. Therefore, compared to other methods, pro-inflammatory factors in the hippocampus had a greater impact on cognitive function, and pro-inflammatory factors in the serum could be cleared in a short period of time or had limited impact on the hippocampus due to the presence of the circulatory system.

The NF-κB signaling pathway is considered as a classical signaling pathway to regulate the inflammation and this signaling pathway is conserved in all mammals (Lin et al. 2020). PKCδ is a multifunctional enzyme to participate in the activation of various signaling pathways. The phosphorylation of PKCδ can stimulate the activation of NF-κB signaling pathway and initiate the downstream inflammatory cascade reactions (Hsu et al. 2001; Li et al. 2019). After being phosphorylated, the p65 would release NF-κB and transfer it into the nucleus (Kawai and Akira 2007). Intranuclear NF-κB would bind to the promoter of the target DNA sequence and enhance the synthesis of various cytokines, including pro-inflammatory factors (Kawai and Akira 2007; Lawrence 2009). In our study, we found a significant increase in the phosphorylation of PKCδ and NF-κB in the hippocampus of mice from the surgery group, which proved the activation of the PKCδ/NF-κB signaling pathway. However, C8-ceramide had ability to inhibit the PKCδ/NF-κB signaling pathway and suppress the inflammatory response induced by surgery. It has showed that C8-ceramide had a bright future in the field of neuro-inflammation treatment.

The bridge of BDNF and inflammatory pathway determines the polarization of microglia. After the exposure of common carotid artery surgery, the immune cells-microglia crosstalk in the CNS induce the polarization of M1 microglia, and the releasing of reactive oxygen species and pro-inflammatory factors, which decrease the synthesis and secretion of BDNF in the hippocampus of mice. We detected the phosphorylation of proteins related to BDNF/TrkB signaling pathway and PKCδ/NF-κB signaling pathway after surgery with/without C8-ceramide treatment. The basis of our study was that microglia were immune cells in the CNS and they could detect changes in the microenvironment in the brain and respond quickly. As we all known, excessive neuroinflammation caused by surgery would damage normal neurons and resulted in the decreasing expression of BDNF and POCD.

Neuro-inflammation only exists in the early stage after surgery, but it may has long-term effect on learning and memory function, due to the fact that excessive inflammatory factors secreted by microglia can damage neurons and the plasticity of synapses and the process of neural regeneration is very slow (Saxena et al. 2019; Fan et al. 2016). Therefore, it will always take a long time to restore damaged neurological function and some patients may even have a lifelong illness. It must be pointed out that the chronic neuro-inflammation significantly inhibit the expression of BDNF which is harmful to the neural regeneration and accelerate the process of POCD (Golia et al. 2019; Lima Giacobbo et al. 2019). However, the BDNF/TrkB signaling pathway, as one of the main molecular mechanisms in the learning and memory process, is important to the synaptic plasticity and the formation of long-term potentiation (LTP) (Parkhurst et al. 2013). The increasing expression of BDNF is beneficial to the anti-inflammation and neural regeneration (Yin et al. 2020). Parkhurst et al. have proved that learning and memory impairment would occur once microglia were completely cleared from the brain (Parkhurst et al. 2013), and it meant that BDNF derived from microglia was important to the cognitive function. Our study has also proved that the increasing expression of BDNF secreted by microglia had ability to improve surgery-induced cognitive impairment. The previous studies have showed that C8-ceramide increased the expression of BDNF in microglia at the dose of 25 μM (Nakajima et al. 2002), which was the same with our study. However, in our study, there was no significant different in the expression of BDNF between the C8-ceramide group and the control group. The possible reason may be that the expression of BDNF was strictly controlled and depended on the microenvironment in the CNS. It is worthy to further study.

Microglia are the resident immune cells in the CNS and can polarize toward pro-inflammatory phenotype (M1 microglia) and anti-inflammatory phenotype (M2 microglia) under specific circumstances (Tang and Le 2016). According to the current theory, the microglia just polarize towards M2 phenotype in a very short period of time and M1 phenotype of microglia are common in plenty of animal neural disease models (Yin et al. 2020). The immunofluorescence staining in our study showed that the microglia in the hippocampus of mice from the surgery group were mainly M1 microglia, while M2 microglia existed in the hippocampus of mice from the C8-ceramide group. Therefore, the application of C8-ceramide may be an effective method to inhibit the neuroinflammation in the CNS and it is an effective method to treat POCD.

Our research found that surgery process significantly down-regulated the BDNF expression and reduced the phosphorylation of TrkB on the 3rd day after surgery. Interestingly, on the 7th day after surgery, the expression of BNDF in the hippocampus of mice was not significantly different among all four groups. We speculated that it might be temporarily up-regulated to the expression of BDNF as compensatory, due to the fact that the behavioral tests on the 8th to 14th days after operation showed that the cognitive function of the mice indeed decreased. Our previous study also found that the expression of BDNF was still significantly lower than that of healthy mice on the 5th day after surgery, and it indicated that the recovery of BDNF secretion started on the 7th day after surgery rather than at the early stage and the recovery of cognitive function was even later. It must be pointed out that we did not measure the concentration of BDNF in the hippocampus of mice on the 14th day after surgery, which provided guidance for our future research. The using of C8-ceramide significantly inhibited or shortened the duration of inflammation in the hippocampus of mice after surgery, which played an important role on the prevention and treatment of POCD.

## Conclusion

In our study, we have proved that surgery could cause neuroinflammation in the CNS to induce POCD and the application of C8-ceramide could enhance the expression of BDNF to suppress neuroinflammation via the regulation of PKCδ/NF-κB signaling pathway for the first time. It has revealed a potential molecular mechanism of surgery-induced POCD and proves a promising treatment method. According to our study, we strongly believe that C8-ceramide will become a star drug to treat surgery-induced POCD and reduce the risk of suffering from POCD in a large population.

## Data Availability

The data set analysed in this study are available from the corresponding author on reasonable request.

## Abbreviations

BCA: Bicinchoninic acid
BDNF: Brain-derived neurotrophic factor
CNS: Central nervous system
DAPI: 4′,6-Diamidino-2-phenylindole
DMEM: Dulbecco’s modified Eagle’s medium
ELISA: Enzyme-linked immunosorbent assay
FBS: Fetal bovine serum
IL-1: Interleukin-1
LPS: Lipopolysaccharide
LSD: Least significant difference
LTP: Long-term potentiation
MAPK: Mitogen-activated protein kinase
MWM: Morris Water Maze
PKC: Protein kinase C
POCD: Postoperative cognitive dysfunction
PS: Penicillin–streptomycin
PVDF: Poly-(vinylidene fluoride)
RT-PCR: Reverse transcription polymerase chain reaction
SDS-PAGE: Sodium dodecyl sulfate–polyacrylamide gel electrophoresis
TNF-ɑ: Tumor necrosis factor-ɑ
